# On the Remedial Power of Spirit of Turpentine in Intestinal Obstruction

**Published:** 1821-09

**Authors:** 


### *On the Remedial Power of Spirit of Turpentine in Intestinal Obstruction.* By Dr. Kinglake.

THE medicinal power of spirit of turpentine has long been recognized, but not with sufficient precision to merit confident reliance on its efficacy. Exhibited to the extent of a drachm at intervals of six or eight hours, it is known occasionally to prove strongly diuretic, denoted at once by an augmented urinary secretion, and by the peculiar odour which it imparts to that fluid. It also seems to imbue, when taken every four or six hours for several successive days, the whole circulating mass of fluids with a terebinthinate smell, as it becomes more or less discoverable in all the various excretions, particularly in the cutaneous and pulmonary exhalations. The odoriferous property of turpentine, no more than that of musk, garlic, assafœtida, amber, and other substances, is not in sufficient concentration to be positively active in any of the situations to which it may be extended; it therefore only evinces that it is capable of extreme tenuity and diffusion.

There are but two instances recorded, in my knowledge, of turpentine having been internally given in obstructed bowels, with a view to its cathartic agency.* Nor, on these occasions, does it appear to have been resorted to from any previous reputation which it possessed of having proved efficacious in purgatively exciting the peristaltic power of the intestines. Its success in the cases referred to was equivocal, in as much as the ultimate cathartic effect that ensued was the general result of various purgative articles, given previously and subsequently to the turpentine that was administered. The patient, however, was at length relieved of a most threatening obstruction, and finally recovered. How far the favourable termination may be justly attributed to either the sole or conjoint aid of turpentine, is a question that can only be solved by additional experience of its efficacy.

The powerful influence which turpentine in clysters occasionally has in exciting an evacuating action of the intestines, and the large doses of that article which had been given for the removal of tape-worm, furnished, in my estimation, an authority for its being tried with safety and probable advantage in an unyielding obstruction of the bowels. Under this impression,

* Vide *London Medical and Physical Journal*, vol. xxxvi. p. 359.

it appeared to me to be applicable, with some prospect of benefit, in the case of a man, of about forty years of age, almost hopelessly suffering from constipated intestines, and for whose relief the most powerful means had been judiciously, but unavailingly, employed during three days. Bleeding, warm bath, blistering, lenient and brisk cathartic medicines, with repeated clysters, were assiduously tried throughout that long interval, without any beneficial effect. The abdomen became much inflated and distended, was extremely painful on pressure, and every thing that was swallowed was, for upwards of the last twenty-four hours, rejected, together with a large quantity of a dark bilious fluid. The pulse was feeble, and at times fluttering; the countenance pale, shrunk, and haggard; the venous circulation was so slow as to induce a livid hue on the extremities: in short, every appearance indicated either the actual existence or speedy approach of destructive gangrene. The circumstances altogether could not be more unfavourable, nor could they portend more imminent danger of life.

Finding, on being consulted, so much unsuccessful endeavour by the most approved modes for obtaining relief, I proposed that the spirit of turpentine should be tried, and directed it in doses of two drachms, conjoined with half an ounce of castor-oil, to be taken every two hours until the bowels should be moved. The first and subsequent doses, to the number of four, remained on the stomach, when a full and complete cathartic effect was produced. No vomiting occurred after the first dose of turpentine; and, in every instance in which it was taken, it induced, to use the patient's own expression, "a constant rumbling and a very pressing sensation on the bowels."*

It deserves to be especially noticed, that neither nausea nor

* This part of the case will be best explained by the following extract of a letter, addressed to me by Mr. Meede, surgeon of North Carey, who attended the patient from an early stage of the complaint to its termination.—" Imagining that you would feel gratified in learning the effect of the medicine prescribed in Mr. Thomas Brinson's case, I most willingly acquaint you with the result. The bolus was taken as soon as it could be conveyed to the patient, and was rejected after about three quarters of an hour.* Then one of the draughts directed by you was given, composed of two drachms of rectified spirit of turpentine and half an ounce of castor-oil, and repeated at intervals of two hours, as ordered. Four of them were taken; and, before the time for giving the fifth arrived, the happy and desired effect was produced by very copious evacuations from the intestines; since which period the patient has been hourly recovering. The medicine sat well on the stomach, without producing the least of either vomiting or nausea. The only sensible effect it appeared to have, was that of occasioning great languor, with a constant rumbling and a very pressing sensation on the bowels. No other observation of importance occurred. I have given you the patient's feelings as described by himself."

* This bolus consisted of powdered scammony and jalap, each half a drachm; and of supertartrite of potass, two drachms.

vomiting was occasioned by the medicine; but that, on the contrary, a quieting influence appeared to result from it, so that, before it operated as a cathartic, it subdued the disordered and rejecting state of stomach which had previously prevailed. The discharges which followed the removal of the obstruction were numerous, very copious, partly scybalous, and extremely fetid.

The enlarged size of the abdomen soon subsided; the tenderness speedily ceased; and, in the course of a few days, the patient was without any other ailment than what had arisen from the agitated and worn state of the general strength. This patient must evidently appear to owe his narrow escape from impending death to the salutary influence of turpentine: no other known medicine could, in my opinion, have equally averted it, in affording the aid which was rendered by this powerful agent.

It has occurred to me to see several deplorable cases of obstructed bowels, in which the necessary relief could not be obtained by any variety of treatment that could be instituted, and in which death, of course, terminated the miserable scene. Had turpentine been employed in these instances, judging from the fairest rules of analogy, it is just to infer that remedial assistance might have been derived from its use. It has never happened to me to witness, not even on the fatal occasions referred to, a case of less promising issue than the one under consideration; and it is my full persuasion that, if it had not been rescued from the difficulties and perils attending it in the way stated, the event must have been mortal.

Another case of obstructed intestines occurred a few weeks since, in which the terebinthinate remedy was again tried. The patient was a man of about fifty years of age, whose general health was tolerably good, but whose bowels were habitually indolent. Unusual constipation had arisen, and which, after a lapse of two days, would not give way to the domestic medicine that had commonly relieved. Recourse was then had to a medical practitioner, who endeavoured to effect a passage by means sufficiently powerful in most cases to have succeeded. An insuperable obstinacy, however, remained. On my being consulted, which I believe was on the third day of the obstruction, the patient was at intervals suffering severe pain; the abdomen was tense, and very uneasy on being pressed; vomiting had taken place, and every thing that was swallowed was, after a short time, rejected. Much general debility and great mental anxiety were manifested. Bleeding, warm bath, cathartics, and enemas of the most active kind, were found incapable of rendering the necessary relief. After fully ascertaining, as should always be done on these occasions, that no hernial derangement had any share in the obstruction, I again proposed that spirit of turpentine should be tried, reciting to the medical gentleman with whom I was associated in consultation the apparent advantage which had resulted from it in the instance before detailed. Conceiving, from the early inefficacy attending its use on the former occasion, that the dose, not being more than two drachms, was too small, it was suggested that it should be given to the extent of half an ounce, with an equal quantity of castor-oil, every hour until the bowels should be sufficiently moved. This medicine was repeatedly taken, but in how many instances my informant does not state. It should seem, however, that it was not retained as in the former case, but, like all other medicine, had been rejected. The patient was then again twice put into a warm-bath, and soon after the bowels were freely moved, and the desired relief was fully obtained. The medical attendant on the patient was inclined to think that the whole of the turpentine which had been taken was not returned by vomiting, but that a portion of it remained, which probably contributed to the purgative effect that was ultimately produced. In this opinion he conceives himself strengthened by the fact of the evacuations having a strong terebinthinate smell.*

This case, it must be owned, is not so unequivocal in its testimony of the purgative agency of turpentine as the former, yet it appears to have been, at least, an important auxiliary in effecting a removal of the obstruction. The case seemed to be of the inveterate kind that is hardly to be overcome, and it is very likely it would not have been surmounted without the exciting influence of turpentine. Unlike what happened in the former case, it was not quietly retained on the stomach, but a considerable portion of it was rejected, which accounts for its more slow and doubtful efficacy in the one instance than in the other.

The great obstacle to the due action of purgative medicine in confined bowels, is the difficulty of retaining it sufficiently long to become operative. The stomach on these occasions is

---

* The medical practitioner who first attended this case is Mr. Mills, surgeon, of Bishop's Lydeard. At my request, he communicated to me the subjoined account of the treatment, which will enable the reader to estimate what share the influence of turpentine had in producing the relief that was finally obtained.—"With respect to the terebinthinate mode of treatment, I cannot give a decisive opinion; as, within an hour after each time of taking the turpentine as directed, it was rejected, but I will not say wholly. Symptoms, however, on the evening of the morning of your seeing the patient, became so severe, that I again had recourse to the warm-bath; and, after twice using it, the desired effect was produced. As the excrementitious substances which came off smelled very strongly of turpentine, I am induced to think that it, in some measure, contributed to the patient's relief."

often so nauseated and irritable, as to be incapable of holding medicine of any description the necessary time for being effective. It is not usual for turpentine to occasion either vomiting or sickness; on the contrary, it seems to allay and soothe an agitated state of stomach. It had that effect very decidedly in the former case; nor does it appear, in the numerous instances in which turpentine has been largely given, either in puerperal affection or for the destruction and expulsion of tape-worm, that it has offended the stomach. It may, therefore, be concluded that it is not its common effect; or, rather, that it is apt to make an impression on that organ that removes nausea, and, as in the case here recited, obviates vomiting.

Spirit of turpentine has been given by Mr. Hartle* in doses of two ounces undiluted, and in the same state ordinarily from two to three ounces by Dr. Fenwick,* in cases of tape-worm; and it is observed that a large quantity passes the bowels more readily, and with less inconvenience, than when more sparingly taken. It is probable that, in the cases stated, an ounce given at a dose would have been at once effective, and that neither two drachms nor half an ounce could sufficiently excite the necessary peristaltic action. It does not appear to possess any specific purgative quality, like aloes, scammony, jalap, &c. but to have the property of energizing, as it were, by its stimulating agency, the contractility of the intestines, so as to enable them to propel and discharge their contents. For the purpose of directing this property more particularly to the peristaltic power of the bowels, it would, perhaps, be more advisable to conjoin it with some known purgative than to exhibit it alone. In the cases above related it was mixed with castor-oil, and it would probably be fully capable of producing the desired opening effect on the bowels in instances of obstruction that would not yield to other means, if given to the extent of an ounce with an equal quantity of castor-oil, repeating it every hour where the occasion might be very urgent, until it should become operative. Much more extended and varied trials of the power of turpentine in overcoming obstinate obstructions of the intestines must be made, before it can be justly regarded as a medicine worthy of entire reliance. All medicines have certain peculiarities in their agency on the human frame, founded on relative circumstances, that can only be detected by adequate experience. The stomach may occasionally be so irritable as not to endure a medicine, which may not only be tolerable, but even grateful, in a less excitable state of that organ. The same may be said of the bowels: they are liable to be variously af-

* Vide *London Medical and Physical Journal*, vol. xl. p. 546.

fected, in different temperaments, by similar medicinal qualities. Such occurrences, however, are exceptions to the general rule, by which a given cause produces a definite effect, and afford no valid objection against the admissibility and salutary powers of the agent that may have been employed.

When turpentine shall be sufficiently understood in all its absolute and relative powers,—when its peculiarities shall be well known, and its general capabilities be fully ascertained,—it will, no doubt, prove to be a medicine of very considerable importance, whether with reference to its active efficiency on emergent occasions, as in intestinal obstruction, or in alterative intentions, to its power of correcting distempered conditions of either the general or visceral excitability of the animal system.

In aid of the cathartic influence which turpentine is designed to exert in obstructed bowels, it may not be amiss to rub it in as a liniment, in conjunction with an equal quantity of olive-oil, over the tumid abdomen usually attending these cases. The strong sympathetic connexion naturally subsisting between the organic structure of the intestines and that of the cutaneous surface of the abdomen, is an excitable medium through which it may be justly conceived that external applications may be rendered salutarily efficient in affections of the bowels. Some effect from briskly rubbing in medicinal agents on the skin might result from absorption; but this way is too circuitous, and too material in the view of its ultimate power, to be relied on with as reasonable a prospect of benefit as that which is afforded by sympathetic agency. In the latter mode, kindred or associated excitability is solely concerned; and, when this property is sufficiently awakened and exerted, its power is forcible, rapid, and direct, and is liable to no mechanical interruption. It may also at the same time be injected, when diluted in the proportion of one ounce to a pint of oat-gruel, as a clyster, repeating it at short intervals until the desired effect be obtained.

The dose of spirit of turpentine and castor-oil, in a purgative intention, in cases of unyielding obstruction of the bowels, whether it be two drachms, half an ounce, or an ounce of each, may be blended together by the intervention of the yolk of an egg; to which may be added an ounce or more of an aqueous vehicle, as may be thought fit. It was in this form that it was directed in the cases which have been here related. Dr. Brenan, who has called the attention of the public to the use of turpentine in even apparently the dying stages of puerperal disease, most frequently gave it alone, but at times diluted. It has also been taken in both forms in cases of tape-worm. Judging from its highly pungent and stimulating flavour, it would seem to be most safe and advisable to dilute it with at least double its quantity of some watery fluid. But, if its remedial efficacy should be found to depend on its concentrated strength, the expediency of reducing it may be questionable, and must await, as in all other instances of imperfect knowledge, the farther testimony of additional and conclusive experience.

*Taunton; July* 16, 1821.

